# RDoC Mechanisms Underlying Bodily Symptoms in Psychiatry: A Scoping Review

**DOI:** 10.2147/PRBM.S601207

**Published:** 2026-10-02

**Authors:** Aude Molinard-Chenu, Vasileios Chytas

**Affiliations:** 1 Department of Psychiatry, Service of Liaison Psychiatry and Crisis Intervention, Geneva University Hospital, Geneva, Switzerland; 2 Psychiatry Division, Hôpital de la Tour, Geneva, Switzerland

**Keywords:** psychosomatic, bodily symptoms, psychiatric disorders, RDoC, scoping review, biopsychosocial

## Abstract

Somatic or bodily symptoms are shared among various psychiatric disorders. Across the mood, anxious, dissociative and psychotic spectra, common and perhaps specific mechanisms for these debilitating symptoms need to be clarified. We propose here a scoping review of studies published until May 2025 focusing on primary research in adult populations about bodily symptoms, psychiatric disorders, and neurobiological mechanisms connecting them, based on the Research Domain Criteria (RDoC) matrix. Our review follows the PRISMA-ScR Checklist and included 73 articles. Studies focusing solely on psychosomatic disorders were excluded. In the included studies, the most represented somatic symptoms were general symptoms such as pain or unspecific somatic symptoms, whereas anxiety and mood disorders accounted for the majority of psychiatric disorders. In terms of mechanistic links between somatic symptoms and psychiatric disorders, somatosensory perception and self-knowledge were the most studied RDoC subconstructs. When combined together, the most common triad associating somatic symptom, RDoC construct and psychiatric disorder was pain - perception - borderline personality disorder, occurring in 11 articles. This work also identifies knowledge gaps in the literature about mechanistic links between somatic symptoms and psychiatric disorders. For example, specific functional syndromes, dissociative disorders and interoception and their interactions are interesting concepts for future primary research studies. Alternatively, the most studied associations suggest topics to be covered by systematic reviews. Altogether, our results emphasize the need for more interdisciplinary research, taking advantage of recent advances in neuroscience that could help to uncover the complex mechanisms at play between psychological and bodily symptoms.

## Introduction

Somatic or bodily symptoms are ubiquitous in medicine^1^ and represent a massive burden of disease and disability worldwide.^2^,^3^ When bodily symptoms are causally associated with a treatable mental or physical disorder, it is imperative to implement a comprehensive diagnostic and therapeutic clinical plan.^4^ However, somatic symptoms can chronically persist without a clear etiology, resulting in a diverse spectrum of symptoms characterized as “functional”.^5–7^ The ICD-11 recently progressed toward a more integrative view of this health issue, introducing the notion of Bodily Distress Disorder (BDD),^8^ which no longer requires these symptoms to be “unexplained by another condition” for a formal diagnosis.^9^,^10^ Furthermore, this nosological entity encompasses a previously diverse range of functional somatic symptoms and somatoform disorders.^11^ There is considerable heterogeneity in the literature concerning the terminology used by researchers to describe somatic expression within psychosomatic medicine. This encompasses, but is not limited to, ICD-based psychosomatic disorders,^12^ functional syndromes,^13^ and specific somatic symptoms.^14^

Many psychiatric conditions are associated with somatic symptoms,^15–17^ adding complexity to their treatment.^18^ Results from the last decades in psychiatry research have highlighted neurobiological underpinnings of many psychiatric disorders^19^,^20^ and since 2009, the Research Domain Criteria (RDoC) initiative has supported novel ways of studying mental disorders via focusing on disruptions of normal body functions.^21^,^22^ As a translational framework for research, it encompasses a matrix that classifies units of analysis (eg genes, behaviors and experimental paradigms) into constructs and subcontstructs (eg loss, perception, attention) that relates to six broader domains (eg cognitive systems, negative valence systems, social processes). Intrinsically, the RDoC framework is predominantly structured around top-down cerebral processes, while bottom-up processes involving extra-cerebral structures - such as the perception of internal stimuli - remain underrepresented among its components.

Individually, the mechanisms underlying certain somatic symptoms are starting to be unraveled. For example, chronic pain most probably results from the integration of different processes^23^ including interoception.^24^ Similarly, psychiatric disorders are increasingly being associated with endophenotypes indicative of neurobiological dysfunctions.^19^,^25^ However, evidence regarding the intersection between somatic symptoms and psychiatric disorder is not clearly available. Here, we propose to summarize the available literature at the crossroads between bodily symptoms, psychiatric disorders and their underlying neurobiological mechanisms. This scoping review aims to highlight key findings and potential knowledge gaps in this field, providing a comprehensive overview of how somatic symptoms may influence or be influenced by psychiatric conditions.

## Methods

### Literature Search, Screening and Selection

Our methodology workflow follows the PRISMA-ScR Checklist^26^ and is summarized in Figure 1.

**Figure 1:**
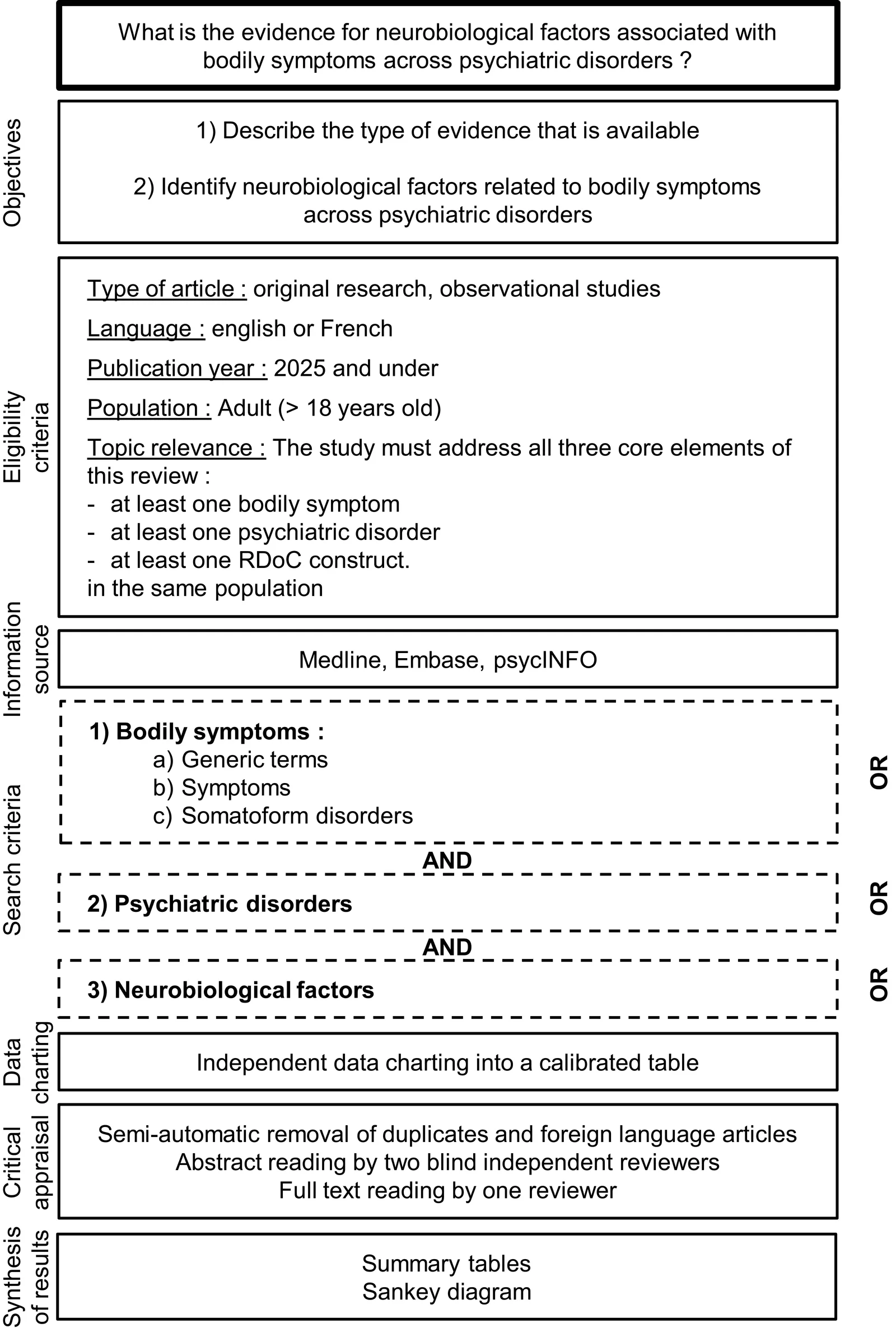
Methodology workflow. This diagram summarizes the methodology of this scoping review, according to the PRISMA-ScR checklist.

Three different information sources were screened (Medline, Embase, psycINFO), using the search queries that are available on SearchRxiv (links are provided below). Databases were first accessed on the 27^th^ of May 2022 and an update search using the same search strings on the same information sources was performed on the 7^th^ of May 2025, covering the period between May 2022 and May 2025. Briefly, the search strategy was to include articles whose titles incorporate terms that refer to a somatic symptom (items from the BDS checklist^14^ and from relevant litterature^13^,^27^ as well as somatoform disorders from ICD-10^12^), a psychiatric disorder based on items from the ICD-10^12^ and items from the RDoC matrix,^28^ version 5, accessed in May 2022. Search strings are available on searchRxiv. Duplicates were excluded using automatic detection features from Mendeley Reference Manager (Elsevier) and Rayyan,^29^ while remaining duplicates were excluded by manual removal based on abstract screening. Inclusion and exclusion criteria were checked by abstract screening performed by two independent reviewers. Consensus about discordant articles was obtained by discussion between reviewers. Full text screening and data charting was performed by a single reviewer. The RDoC matrix, version 5, accessed between 2022 and 2026 was used for this analysis. When an article addressed multiple bodily symptoms, psychiatric disorders, or RDoC constructs, they were all recorded in the data table Studies focusing on a psychosomatic disorder where a somatic symptom and a psychiatric disorder overlap (eg somatoform disorder, conversion disorder) as the only psychiatric disorder were excluded, because our goal was to specifically investigate somatic symptoms as comorbidities to traditional psychiatric disorders rather than primary functional diagnoses Pain located in more than one location, fibromyalgia and unspecific pain were grouped as “pain” and mentions of general somatic symptoms were classified as “somatic symptoms (unspecified)”. Outcome measures were classified based on the ISPOR Clinical Outcome Assessment framework.^30^

### Data Analysis and Graphical Representation

Data tables were collected and analyzed using Microsoft Excel. The Sankey diagram was generated in Jupyter Notebook.^31^ The corresponding python code, as well as all data tables are available upon request to the authors. Graphical adaptations of the figures were done using Microsoft Powerpoint or Inkscape.

## Results

After a thorough selection process, 73 articles were included in the study. The study flowchart is summarized in Figure 2. The list of included articles,^32–104^ along with their extracted content, is provided in Supplementary File 1A–I. Most articles were observational cross-sectional prospective studies (n=69), but 2 retrospective studies and 2 case reports were also included. 55 articles studied unspecified adult populations, but studies on gender-specific somatic symptoms such as endometriosis or premature ejaculation included only females in the former (n=14) or males in the latter (n=2). Germany was the leading country where studied populations were recruited (n=19), followed by the USA (n=15) and Turkey (n=6). Included articles were published between 1989 and 2025, while 70% of the included articles (n=51) were published after 2010.

**Figure 2:**
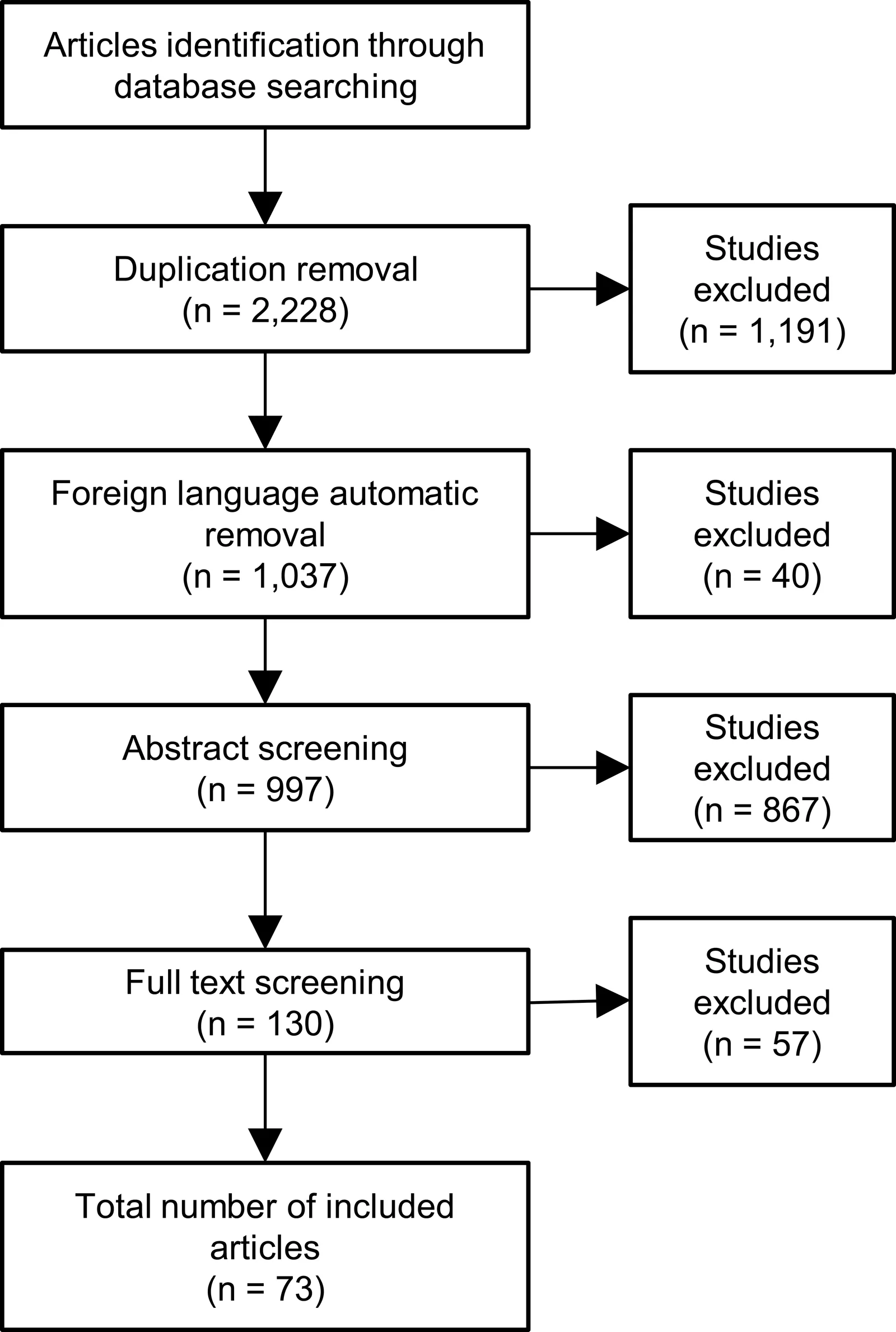
Study flowchart. This flow chart reports the number of excluded articles at each stage of the selection process, leading to a total of 73 included articles.

In total, 75 somatic symptoms were reported, most of them referring to general symptoms such as pain (n=33) or broad somatic symptoms (n=34). These symptoms were assessed by heterogeneous scales or sometimes a subscale of a clinical scale that was not designed to assess somatic symptoms. However, few studies were examining gastro-intestinal system symptoms (n=4) and genito-urinary system symptoms (n=4) (Table 1).

**Table 1:** Bodily Symptoms Represented in the Included Articles

Bodily Symptom	Number of Occurrences	%
Pain	33	44.0
Somatic symptoms (unspecified)	34	45.3
Gastro-Intestinal system symptoms	4	5.3
Genito-urinary system symptoms	4	5.3
**Total**	**75**	**100.0**

**Notes**: The 73 included articles covered 75 bodily symptoms, grouped into 4 main categories: pain, unspecific somatic symptoms, gastro-intestinal system symptoms and genito-urinary system symptoms.

105 psychiatric conditions were identified in our sample (Table 2), anxiety and fear-related disorders being the most studied psychiatric disorders (n=40), followed by mood disorders (n=27), personality disorders (n=14) and psychotic disorders (n=9). Other psychiatric disorders were also mentioned in some articles, such as stress-related disorders (n=6), eating disorders (n=4), depersonalization derealization disorder (n=1), substance use disorders (n=3) and autism (n=1).

**Table 2:** Psychiatric Disorders Represented in the Included Articles

Psychiatric Disorder (ICD-11)		Number of Occurrences	%
Anxiety or fear-related disorders	Generalized Anxiety Disorder	9	8.6
	Panic Disorder	5	4.8
	Illness anxiety	1	1.0
	Agoraphobia	1	1.0
	Anxiety Disorders (unspecified)	23	21.9
	Obsessive compulsive disorder	1	1.0
	**Subtotal**	**40**	**38.1**
Mood disorders	Depressive disorders	23	21.9
	Bipolar disorder	1	1.0
	Mood disorders (unspecified)	3	2.9
	**Subtotal**	**27**	**25.7**
Personality disorders	Borderline Personality Disorder	11	10.5
	Personality disorders (unspecified)	3	2.9
	**Subtotal**	**14**	**13.3**
Psychotic disorders	Schizophrenia	8	7.6
	Psychotic disorders (unspecified)	1	1.0
	**Subtotal**	**9**	**8.6**
Stress-related disorders	Post-traumatic stress disorder	4	3.8
	Complex Post-Traumatic disorder	1	1.0
	Adjustment disorder	1	1.0
	**Subtotal**	**6**	**5.7**
Eating disorders	Bulimia Nervosa	2	1.9
	Anorexia Nervosa	1	0.9
	Eating disorders (unspecified)	1	0.9
	**Subtotal**	**4**	**3.8**
Other	Substance use disorders	3	1.0
	Depersonalization derealisation disorder	1	2.9
	Autism	1	1.0
	**Subtotal**	**5**	**4.8**
**Total**		**105**	**100.0**

**Notes**: The 73 included articles covered 105 psychiatric disorders, distributed across different ICD-11 categories for psychiatric disorders.

Clinical cases were defined by ClinRO (57.5%) and PRO (42.5%) for psychiatric disorders, whereas for bodily symptoms, most clinical cases were defined by PRO (86.3%). Neurobiological processes were assessed by PRO (55.4%), PerfO (31.1%), Biomarkers (10.8%) and ClinRO (2.7%). fMRI was the most used biomarker, used in 5 articles. Detailed results about the types of measurements are available in Supplementary File 1I.

Regarding neurobiological processes, somatosensory perception was the most studied RDoC subconstruct (n=25), followed by self-knowledge (n=13) and potential threat (n=10). Other RDoC constructs and subconstructs are detailed in Table 3.

**Table 3:** Neurobiological Processes Represented in the Included Articles

RDoC Domains	RDoC Constructs	RDoC Subconstructs	Number of Occurrences	%
Cognitive systems	Perception	Somatosensory Perception	26	29.5
	Attention	NA	5	5.7
	Declarative memory	NA	2	2.3
	Working memory	NA	1	1.1
	Language	NA	1	1.1
		**Subtotal**	**34**	**39.1**
Negative Valence Systems	Potential Threat (“Anxiety”)	NA	10	11.5
	Sustained Threat	NA	8	9.2
	Acute Threat (“Fear”)	NA	4	4.6
	Loss	NA	2	2.3
		**Subtotal**	**24**	**27.6**
Social processes	Perception and understanding of self	Agency	1	1.1
		Self-knowledge	13	14.9
	Affiliation and Attachment	NA	7	8.0
	Social Communication	NA	1	1.1
	Perception and Understanding of Others	Understanding Mental States	2	2.3
		**Subtotal**	**24**	**27.6**
Arousal and Regulatory Systems	Arousal	NA	3	3.4
	Sleep-Wakefulness	NA	1	1.1
	Circadian Rhythms	NA	1	1.1
		**Subtotal**	**5**	**5.7**
**Total**			**87**	**100.0**

**Notes**: The 73 included articles covered 87 RDoC constructs and subconstructs, distributed across 4 domains.

Figure 3 is a Sankey diagram illustrating how bodily symptoms, RDoC constructs and psychiatric disorders are interconnected within the analyzed articles. The underlying data are available in Supplementary File 1J. The most common triad was pain-perception-borderline personality disorder, occurring in 10 articles, followed by pain-perception-schizophrenia, occurring in 7 articles. Most neurobiological mechanisms were common to various bodily symptoms and psychiatric disorders, whereas “perception” was specifically associated with pain as a bodily symptom, but it was associated with many psychiatric disorders. Only one article focused on a specific type of pain (chest pain) and was then individualized for the visualization. Interestingly, it is the only bodily symptom that is associated to the RDoC construct “loss”. Some papers included information on more than one bodily symptom, psychiatric disorder or RDoC construct/subconstruct, hence there are more associations than the total number of included articles.

**Figure 3:**
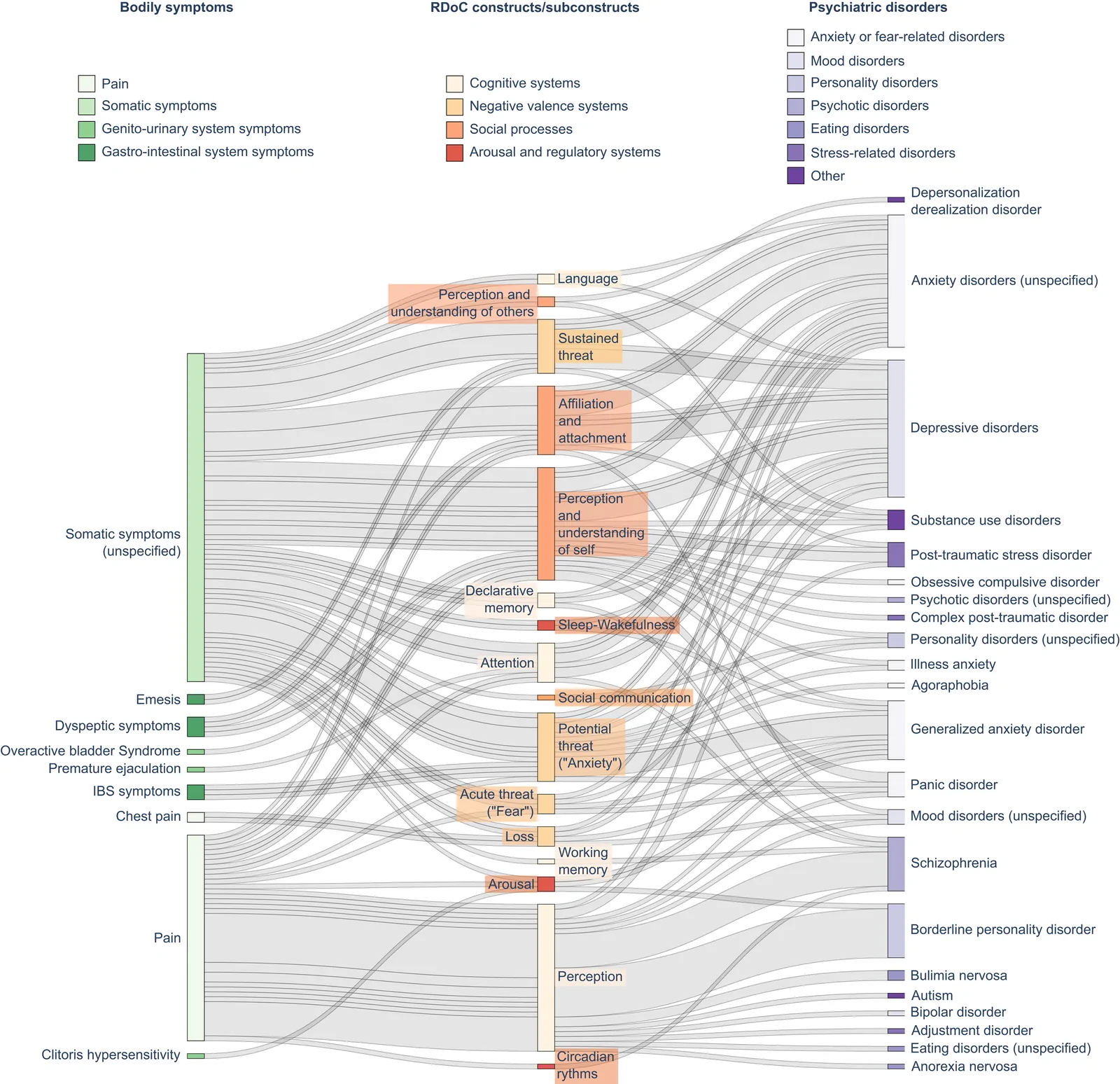
Triads of bodily symptom – RDoC construct/subconstruct – psychiatric disorder in the included articles. Sankey diagram illustrating the connections between bodily symptoms and psychiatric disorders through RDoC constructs and subconstructs in the included articles.

**Abbreviations**: RDoC, Research Domain Criteria, IBS, Irritable bowel syndrome.

## Discussion

This scoping review identified a growing body of research examining the interplay between bodily symptoms and psychiatric disorders through mechanistic processes that were mapped on the RDoC matrix. While some systematic reviews were focusing on specific links between bodily symptoms, neurobiological processes and psychiatric disorders,^105–109^ to our knowledge we present here the first trial to compile primary literature on this broad topic in a single scoping review.

Most studies were referring to general somatic symptoms instead of specific syndromes or symptoms. This phenomenon could be partially explained by our search methodology -principally based on general symptoms from the BDS checklist - but it is worth noting that common psychosomatic syndromes included in our search strategy (eg fatigue syndromes, hyperventilation syndrome or multiple chemical sensitivity) did not yield any publication. However, it is now debated whether these syndromes should still be separated rather than grouped into one diagnostic entity: BDD.^13^ Given the hypothesis that bodily perceptions of emotions are conserved throughout populations,^110^ future studies should aim to delineate more specific somatic patterns that are associated with different psychiatric disorders.

Our results demonstrate that anxiety and mood disorders account for more than 50% of studied psychiatric disorders in the context of a mechanistic study related to somatic symptoms. Noticeably, few studies cover neurodevelopmental disorders, although mind–body interactions are emerging as novel mechanisms in these conditions.^111^ Dissociative symptoms are shared by various nosologic entities and commonly associated with bodily distress disorder.^112^ In our corpus, disorders typically presenting with dissociative symptoms such as PTSD-related disorders and depersonalization-derealization disorder only account for 6/105 psychiatric disorders, highlighting the need to further explore neurobiological underpinnings of somatic manifestations of dissociative disorders. Also characterized by dissociative symptoms, borderline personality disorder is well represented in our corpus, studied in association with pain and perception. Given the fact that self-harming behavior is a core-symptom of borderline personality disorder, it is not surprising that this triad emerges as the most frequently studied in our review. In line with this finding, specific literature reviews are emerging on this topic,^113–115^ suggesting an interesting subject to be covered by a meta-analysis or a broader topic review.

RDoC is a widely used framework that systematically catalogues neurobiological constructs in the context of mental health. It has been actively promoted by governmental health agencies, such as the National Institute of Mental Health (NIMH) in the United States, which have made the allocation of research grants conditional upon the integration of projects within the RDoC framework. The observed increase in the number of included publications since 2010 may partly be explained by this prioritization of mechanistically driven research in psychiatry, but it also reflects a broader trend observed across scientific literature over the past decades.^116^ The RDoC matrix was chosen to map neurobiological mechanisms using a reductionist strategy. However, it has not been updated since 2019 and it excludes interesting processes such as gut–brain interactions^117^ that could be covered by a complementary review. “Somatosensory perception” and “perception and understanding of self” represent almost half of the neurobiological constructs that have been correlated to psychiatric disorders and somatic symptoms in our sample. Unsurprisingly, somatosensory perception was exclusively related to pain as a somatic symptom, whereas self-perception is a more heterogeneous concept that incorporates well-known concepts in psychosomatic medicine such as alexithymia and interoception.^108^,^109^,^118^ Further primary research should indeed take advantage of the increasing knowledge about interoceptive processes,^119^ in order to characterize more precisely its implication in specific psychosomatic interactions. Moreover, a recent review also suggests that interoceptive technologies should be studied as therapeutic interventions in psychiatric conditions.^120^ Taking a broader view, computational psychiatry and psychosomatics^121^ stands as a promising integrative discipline, uniquely positioned to inform and stimulate primary research through computational modeling approaches.

On the other side, affiliation and attachment represents an understudied (n = 7/87) psychological mechanism in the context of borderline personality disorder and somatic symptoms, given that dysfunctional attachment styles are known to contribute to the emergence of this disorder.^122^ Attentional processes should also raise more concern in the interface between psychiatric disorders and somatic symptoms, representing only 8/87 RDoC constructs in our review. Interestingly, sensorimotor systems are not represented in our corpus, whereas it is known to be affected in functional neurological syndrome.^123^ This may partly reflect the domain’s novelty within the framework, as it represents the most recent addition to the RDoC matrix, integrated in 2019,^124^ leaving comparatively less time for a body of research to accumulate.

In summary, our scoping review identifies interesting knowledge gaps that need to be addressed by further specific primary research about potential mechanisms mediating the interaction between somatic symptoms and psychiatric disorders (Table 4). Furthermore, it recognizes areas of inquiry that possess a sufficient body of literature to warrant systematic reviews.

**Table 4:** Knowledge Gaps

Bodily Symptoms	RDoC Constucts/Subconstructs	Psychiatric Disorders
– Fatigue syndromes (chronic fatigue syndrome, postviral chronic fatigue syndrome) – Globus syndrome – Multiple chemical sensitivity	– Interoception – Affiliation and attachment – Attention – Sensorimotor systems	– Neurodevelopmental disorders (eg autism spectrum disorder) – Disorders associated with dissociative symptoms (PTSDs, depersonalination-derealization disorder)

**Notes**: This table summarizes research areas that were not adequately covered, according to the results of this scoping review, indicating knowledge gaps that future research should address.

Further investigation into these subjects and their interactions could reveal important insights into how neurobiological processes influence both psychiatric and somatic symptomatology. Our findings also reveal a significant gap concerning the populations examined. Further research should prioritize older adults, as somatic symptoms frequently co-occur with psychiatric conditions in this demographic category.^125^ Specifically, a recent review^126^ highlights how risk factors accumulate across the lifespan in the context of nociplastic pain, paving the way for future studies about the relationship between mental disorders and nociplastic pain in older adults. It is a recognized issue that older adults are not sufficiently represented in clinical research.^127^ Furthermore, the intricate and multifaceted characteristics of geriatric conditions might not fully fit within the RDoC framework, which emphasizes the developmental trajectories of mental health conditions.^22^

In addition to the previously mentioned limitations regarding the RDoC matrix’s reductionist impact on the identification of neurobiological processes, our study has further constraints. Firstly, even if each included article focused on specific psychiatric conditions, it is important to acknowledge the variability in how these conditions are evaluated across studies. Some studies use definitive diagnoses according to international standards and clinician evaluations, while others rely on self-reported questionnaires for psychiatric symptoms. This variability may compromise the reliability of mechanistic associations with RDoC constructs by conflating properly diagnosed patients with individuals presenting with psychological symptoms. However, in the context of somatic complaints, embracing patient subjectivity by collecting PROs seems paramount. Secondly, each article that was included in this review identifies a relationship between somatic symptoms, psychiatric disorders, and RDoC constructs; however, this does not necessarily imply a causal relationship or a robust statistical association.

## Conclusion

The most common triad associating somatic symptom, RDoC construct and psychiatric disorder was pain-perception-borderline personality disorder, followed by pain-perception-schizophrenia. Altogether, this scoping review sheds light to a growing area of research characterized by its diverse and scattered nature, highlighting the critical need for interdisciplinary research in psychosomatic medicine. There is especially need for a shift from cross-sectional associative studies toward longitudinal cohorts or neurobiologically informed experimental/interventional designs. More coordinated efforts could help to better understand the complex interactions between psychological and bodily symptoms, ultimately aiming to improve diagnostic approaches and treatment strategies.

## Data Availability

Search strings are available on searchRxiv.^128–130^ All data generated or analysed during this study are available upon request to the corresponding author.

## References

[1] Green LA, Fryer GE, Yawn BP, Lanier D, Dovey SM. The ecology of medical care revisited. *N Engl J Med*. 2001;344(26):2021–14. doi:10.1056/NEJM200106283442611 11430334

[2] Barsky AJ, Orav EJ, Bates DW. Somatization increases medical utilization and costs independent of psychiatric and medical comorbidity. *Arch Gen Psychiatry*. 2005;62(8):903–910. doi:10.1001/ARCHPSYC.62.8.903 16061768

[3] Rice ASC, Smith BH, Blyth FM. Pain and the global burden of disease. *Pain*. 2016;157(4):791–796. doi:10.1097/J.PAIN.0000000000000454 26670465

[4] Roenneberg C, Sattel H, Schaefert R, Henningsen P, Hausteiner-Wiehle C. Functional somatic symptoms. *Dtsch Arztebl Int*. 2019;116(33–34):553. doi:10.3238/arztebl.2019.0553 PMC6794707 31554544

[5] Henningsen P. Fear of flying: relying on the absence of organic disease in functional somatic symptoms. *J Psychosomatic Res*. 2016;88:59. doi:10.1016/j.jpsychores.2016.07.007 27455913

[6] Brown RJ. Introduction to the special issue on medically unexplained symptoms: background and future directions. *Clinic Psychol Rev*. 2007;27(7):769–780. doi:10.1016/j.cpr.2007.07.003 17707564

[7] Stone J, Wojcik W, Durrance D, et al. What should we say to patients with symptoms unexplained by disease? The “number needed to offend. *Br Med J*. 2002;325(7378):1449–1450. doi:10.1136/bmj.325.7378.1449 PMC139034 12493661

[8] Budtz-Lilly A, Schröder A, Rask MT, Fink P, Vestergaard M, Rosendal M. Bodily distress syndrome: a new diagnosis for functional disorders in primary care? Clinical presentation, diagnosis, and management. *BMC Family Pract*. 2015;16(1):1–10. doi:10.1186/S12875-015-0393-8/TABLES/2 PMC4681035 26669977

[9] Burton C, Fink P, Henningsen P, Löwe B, Rief W. Functional somatic disorders: discussion paper for a new common classification for research and clinical use. *BMC Med*. 2020;18(1):1–7. doi:10.1186/S12916-020-1505-4/FIGURES/1 PMC7052963 32122350

[10] Löwe B, Levenson J, Depping M, et al. Somatic symptom disorder: a scoping review on the empirical evidence of a new diagnosis. *Psychological Medicine*. 2022;52(4):632–648. doi:10.1017/S0033291721004177 PMC8961337 34776017

[11] Fink P, Schröder A. One single diagnosis, bodily distress syndrome, succeeded to capture 10 diagnostic categories of functional somatic syndromes and somatoform disorders. *J Psychosomatic Res*. 2010;68(5):415–426. doi:10.1016/J.JPSYCHORES.2010.02.004 20403500

[12] ICD-10 version: 2019. ICD-10 - World Health Organization. 2019. Available from: https://icd.who.int/browse10/2019/en. Accessed March 8, 2022.

[13] Wessely S, Nimnuan C, Sharpe M. Functional somatic syndromes: one or many? *Lancet*. 1999;354:9182):936–939. doi:10.1016/S0140-6736(98)08320-2 10489969

[14] Budtz-Lilly A, Fink P, Ørnbøl E, et al. A new questionnaire to identify bodily distress in primary care: the ‘BDS checklist. *J Psychosomatic Res*. 2015;78(6):536–545. doi:10.1016/j.jpsychores.2015.03.006 25818346

[15] Bekhuis E, Schoevers RA, Van Borkulo CD, Rosmalen JGM, Boschloo L. The network structure of major depressive disorder, generalized anxiety disorder and somatic symptomatology. *Psychological Medicine*. 2016;46(14):2989–2998. doi:10.1017/S0033291716001550 27523095

[16] de Vroege L, de Heer EW, Van der Thiel E, Van den Broek KC, Van Eck Van der Sluijs JF, Van der Feltz-Cornelis CM. Type D Personality. Concomitant Depressive and Anxiety Disorders, and Treatment Outcomes in Somatic Symptom and Related Disorders: an Observational Longitudinal Cohort Study. *Front Psychiatry*. 2019;10. doi:10.3389/fpsyt.2019.00417 PMC6611395 31316400

[17] Edgcomb JB, Tseng CH, Kerner B. Medically unexplained somatic symptoms and bipolar spectrum disorders: a systematic review and meta-analysis. *J Affective Disorders*. 2016;204:205–213. doi:10.1016/J.JAD.2016.06.029 27371906

[18] Van Eck van der Sluijs JF, de Vroege L, Van Manen AS, Rijnders CAT, Van der Feltz-Cornelis CM. Complexity assessed by the INTERMED in patients with somatic symptom disorder visiting a specialized outpatient mental health care setting: a Cross-sectional Study. *Psychosomatics*. 2017;58(4):427–436. doi:10.1016/J.PSYM.2017.02.008 28347505

[19] Cuthbert BN, Morris SE. Evolving concepts of the schizophrenia spectrum: a research domain criteria perspective. *Front Psychiatry*. 2021;12:641319. doi:10.3389/FPSYT.2021.641319/BIBTEX PMC7947312 33716834

[20] Goldstein-Piekarski AN, Ball TM, Samara Z, et al. Mapping neural circuit biotypes to symptoms and behavioral dimensions of depression and anxiety. *Biol Psychiatry*. 2022;91(6):561–571. doi:10.1016/J.BIOPSYCH.2021.06.024/ATTACHMENT/568D3837-7BAA-4B6C-BF2E-6CA901AD080B/MMC2.XLSX PMC9511971 34482948

[21] Cuthbert BN, Insel TR. Toward the future of psychiatric diagnosis: the seven pillars of RDoC. *Bmc Med*. 2013;11(1):126. doi:10.1186/1741-7015-11-126 PMC3653747 23672542

[22] Morris SE, Sanislow CA, Pacheco J, Vaidyanathan U, Gordon JA, Cuthbert BN. Revisiting the seven pillars of RDoC. *BMC Med*. 2022;20(1):1–11. doi:10.1186/S12916-022-02414-0/FIGURES/1 PMC9245309 35768815

[23] Eckert AL, Pabst K, Endres DM. A Bayesian model for chronic pain. *Front Pain Res*. 2022;3:152. doi:10.3389/FPAIN.2022.966034 PMC9595216 36303889

[24] Solcà M, Park HD, Bernasconi F, Blanke O. Behavioral and neurophysiological evidence for altered interoceptive bodily processing in chronic pain. *NeuroImage*. 2020;217. doi:10.1016/J.NEUROIMAGE.2020.116902 32438047

[25] Van Oort J, Llera A, Kohn N, et al. Brain structure and function link to variation in biobehavioral dimensions across the psychopathological continuum. *eLife*. 2023;12:e85006. doi:10.7554/eLife.85006 PMC10519708 37334965

[26] Tricco AC, Lillie E, Zarin W, et al. PRISMA extension for scoping reviews (PRISMA-ScR): checklist and explanation. *Ann Internal Med*. 2018;169(7):467–473. doi:10.7326/M18-0850 30178033

[27] Kohlmann S, Löwe B, Shedden-Mora MC. Health care for persistent somatic symptoms across Europe: a qualitative evaluation of the EURONET-SOMA expert discussion. *Front Psychiatry*. 2018;9:646. doi:10.3389/FPSYT.2018.00646/BIBTEX PMC6292948 30581394

[28] RDoC matrix. Available form: https://www.nimh.nih.gov/research/research-funded-by-nimh/rdoc/constructs/rdoc-matrix. Accessed September 25, 2026.

[29] Ouzzani M, Hammady H, Fedorowicz Z, Elmagarmid A. Rayyan-a web and mobile app for systematic reviews. *Syst Rev*. 2016;5(1):1–10. doi:10.1186/S13643-016-0384-4/FIGURES/6 PMC5139140 27919275

[30] Walton MK, Powers JH, Hobart J, et al. Clinical outcome assessments: conceptual foundation—report of the ISPOR clinical outcomes assessment – emerging good practices for outcomes research task force. *Value Health*. 2015;18(6):741–752. doi:10.1016/j.jval.2015.08.006 PMC4610138 26409600

[31] Thomas K, Benjamin R-K, Fernando P, et al. Jupyter Notebooks - a publishing format for reproducible computational workflows. In: *Positioning and Power in Academic Publishing: Players, Agents and Agendas*. IOS Press; 2016. doi:10.3233/978-1-61499-649-1-87

[32] Son SH, Jo H, Rim HD, et al. A comparative study on alexithymia in depressive, somatoform, anxiety, and psychotic disorders among Koreans. *Psychiatry Invest*. 2012;9(4):325–331. doi:10.4306/pi.2012.9.4.325 PMC3521107 23251195

[33] Ludäscher P, Greffrath W, Schmahl C, et al. A cross-sectional investigation of discontinuation of self-injury and normalizing pain perception in patients with borderline personality disorder. *Acta psychiatrica Scandinavica*. 2009;120(1):62–70. doi:10.1111/j.1600-0447.2008.01335.x 19133877

[34] Vijay KG, Avasthi A, Grover S. A study of worry and functional somatic symptoms in generalized anxiety disorder. *Asian J Psych*. 2014;11:50–52. doi:10.1016/j.ajp.2014.05.011 25453697

[35] De Berardis D, Campanella D, Gambi F, et al. Alexithymia, fear of bodily sensations, and somatosensory amplification in young outpatients with panic disorder. *Psychosomatics*. 2007;48(3):239–246. doi:10.1176/appi.psy.48.3.239 17478593

[36] Murakami H, Tamasawa N, Yamashita M, et al. Altered pain perception in schizophrenia. *Lancet*. 2010;375:9717):864. doi:10.1016/S0140-6736(09)62061-4 20206779

[37] Maunder RG, Hunter JJ, Atkinson L, et al. An attachment-based model of the relationship between childhood adversity and somatization in children and adults. *Psychosomatic Med*. 2017;79(5):506–513. doi:10.1097/PSY.0000000000000437 27941580

[38] Michal M, Adler J, Reiner I, et al. Association of neglect-like symptoms with anxiety, somatization, and depersonalization in complex regional pain syndrome. *Pain Med*. 2017;18(4):764–772. doi:10.1093/pm/pnw214 27605590

[39] Neumann E, Sattel H, Gündel H, Henningsen P, Kruse J. Attachment in romantic relationships and somatization. *J Nerv Mental Dis*. 2015;203(2):101–106. doi:10.1097/NMD.0000000000000241 25594785

[40] Orme W, Kapoor S, Frueh BC, Allen JG, Fowler JC, Madan A. Attachment style mediates the relationship between trauma and somatic distress among individuals with serious mental illness. *Psychiatry*. 2021;84(2):150–164. doi:10.1080/00332747.2021.1930427 34293279

[41] Lautenbacher S, Pauli P, Zaudig M, Birbaumer N. Attentional control of pain perception: the role of hypochondriasis. *J Psychosomatic Res*. 1998;44(2):251–259. doi:10.1016/s0022-3999(97)00214-6 9532554

[42] Kumar V, Avasthi A, Grover S. Correlates of worry and functional somatic symptoms in generalized anxiety disorder. *Indust psych j*. 2019;28(1):29–36. doi:10.4103/ipj.ipj_31_19 PMC6929227 31879444

[43] Bekrater-Bodmann R, Chung BY, Richter I, et al. Deficits in pain perception in borderline personality disorder: results from the thermal grill illusion. *Pain*. 2015;156(10):2084–2092. doi:10.1097/j.pain.0000000000000275 PMC4770381 26098439

[44] Schloss N, Shabes P, Kuniss S, et al. Differential perception of sharp pain in patients with borderline personality disorder. *Eur J Pain*. 2019;23(8):1448–1463. doi:10.1002/ejp.1411 31034113

[45] Lemche E, Surguladze SA, Brammer MJ, et al. Dissociable brain correlates for depression, anxiety, dissociation, and somatization in depersonalization-derealization disorder. *CNS Spectrums*. 2016;21(1):35–42. doi:10.1017/S1092852913000588 24059962

[46] Newby JM, Hobbs MJ, Mahoney AEJ, Wong SK, Andrews G. DSM-5 illness anxiety disorder and somatic symptom disorder: comorbidity, correlates, and overlap with DSM-IV hypochondriasis. *J Psychosomatic Res*. 2017;101:31–37. doi:10.1016/j.jpsychores.2017.07.010 28867421

[47] Leibbrand R, Hiller W, Fichter MM. Effect of comorbid anxiety, depressive, and personality disorders on treatment outcome of somatoform disorders. *Comprehensive psychiatry*. 1999;40(3):203–209. doi:10.1016/s0010-440x(99)90004-4 10360615

[48] Schmidt NB, Cook JH. Effects of anxiety sensitivity on anxiety and pain during a cold pressor challenge in patients with panic disorder. *Behav Res Ther*. 1999;37(4):313–323. doi:10.1016/s0005-7967(98)00139-9 10204277

[49] Ludäscher P, Bohus M, Lieb K, Philipsen A, Jochims A, Schmahl C. Elevated pain thresholds correlate with dissociation and aversive arousal in patients with borderline personality disorder. *Psychiatry Res*. 2007;149(1–3):291–296. doi:10.1016/j.psychres.2005.04.009 17126914

[50] Malejko K, Huss A, Schönfeldt-Lecuona C, Braun M, Graf H. Emotional components of pain perception in borderline personality disorder and major depression-a repetitive peripheral magnetic stimulation (rPMS) study. *Brain Sciences*. 2020;10(12). doi:10.3390/brainsci10120905 PMC7761125 33255481

[51] Kani HT, Dural U, Sakalli Kani A, et al. Evaluation of depression, anxiety, alexithymia, attachment, social support and somatization in functional dyspepsia. *Psych Clin Psychopharmacol*. 2019;29(1):45–51. doi:10.1080/24750573.2018.14

[52] Mussgay L, Klinkenberg N, Rüddel H. Heart beat perception in patients with depressive, somatoform, and personality disorders. *J Psychophysiol*. 1999;13(1):27–36.

[53] Kivrak Y, Yagci I, Yagci HP, Tasdelen Y, Asoglu M. Hyperemesis gravidarum is associated with childhood trauma, depression, trait anxiety, and somatization: a case- control study. *Dusunen Adam*. 2018;31(2):177–186. doi:10.5350/JPN2018310205

[54] Liu S, Zhang X, Wang Q, et al. Increased attentional network activity in premature ejaculation patients with anxiety revealed by resting-state functional magnetic resonance imaging. *Eur J Neurosci*. 2021;54(4):5417–5426. doi:10.1111/ejn.15402 34327757

[55] Failla MD, Gerdes MB, Williams ZJ, Moore DJ, Cascio CJ. Increased pain sensitivity and pain-related anxiety in individuals with autism. *Pain Reports*. 2020;5(6):e861. doi:10.1097/PR9.0000000000000861 PMC7676593 33235944

[56] Nacak Y, Morawa E, Tuffner D, Erim Y. Insecure attachment style and cumulative traumatic life events in patients with somatoform pain disorder: a cross-sectional study. *J Psychosomatic Res*. 2017;103:77–82. doi:10.1016/j.jpsychores.2017.10.003 29167050

[57] Bär KJ, Berger S, Schwier C, Wutzler U, Beissner F. Insular dysfunction and descending pain inhibition in anorexia nervosa. *Acta psychiatrica Scandinavica*. 2013;127(4):269–278. doi:10.1111/j.1600-0447.2012.01896.x 22747702

[58] Sugaya N, Yoshida E, Yasuda S, et al. Irritable bowel syndrome, its cognition, anxiety sensitivity, and anticipatory anxiety in panic disorder patients. *Psych Clin Neurosci*. 2013;67(6):397–404. doi:10.1111/pcn.12069 23890055

[59] Bobevski I, Kissane DW, Vehling S, McKenzie DP, Glaesmer H, Mehnert A. Latent class analysis differentiation of adjustment disorder and demoralization, more severe depressive and anxiety disorders, and somatic symptoms in patients with cancer. *Psycho-Oncology*. 2018;27(11):2623–2630. doi:10.1002/pon.4761 29761579

[60] Waldinger RJ, Schulz MS, Barsky AJ, Ahern DK. Mapping the road from childhood trauma to adult somatization: the role of attachment. *Psychosomatic Med*. 2006;68(1):129–135. doi:10.1097/01.psy.0000195834.37094.a4 16449423

[61] Wingenfeld K, Terfehr K, Meyer B, Löwe B, Spitzer C. Memory bias for emotional and illness-related words in patients with depression, anxiety and somatization disorders: an investigation with the directed forgetting task. *Psychopathology*. 2012;46(1):22–27. doi:10.1159/000338609 22869355

[62] Papadopoulou M, Papapostolou A, Bakola E, et al. Neurophysiological and ultrasonographic comparative study of autonomous nervous system in patients suffering from fibromyalgia and generalized anxiety disorder. *Neurological Sci*. 2022;43(4):2813–2821. doi:10.1007/s10072-021-05606-3 34557968

[63] Guieu R, Samuélian JC, Coulouvrat H. Objective evaluation of pain perception in patients with schizophrenia. *British J Psychiatry*. 1994;164(2):253–255. doi:10.1192/bjp.164.2.253 8173831

[64] Greenberg J, Burns JW. Pain anxiety among chronic pain patients: specific phobia or manifestation of anxiety sensitivity? *Behav Res Ther*. 2003;41(2):223–240. doi:10.1016/s0005-7967(02)00009-8 12547382

[65] Bohus M, Limberger M, Ebner U, et al. Pain perception during self-reported distress and calmness in patients with borderline personality disorder and self-mutilating behavior. *Psychiatry Res*. 2000;95(3):251–260. doi:10.1016/s0165-1781(00)00179-7 10974364

[66] Atik L, Konuk N, Akay O, Ozturk D, Erdogan A. Pain perception in patients with bipolar disorder and schizophrenia. *Acta Neuropsychiatr*. 2007;19(5):284–290. doi:10.1111/j.1601-5215.2007.00193.x 26952940

[67] Blumensohn R, Ringler D, Eli I. Pain perception in patients with schizophrenia. *J Nerv Mental Dis*. 2002;190(7):481–483. doi:10.1097/00005053-200207000-00011 12142852

[68] Stein D, Kaye WH, Matsunaga H, et al. Pain perception in recovered bulimia nervosa patients. *Int J Eating Disord*. 2003;34(3):331–336. doi:10.1002/eat.10164 12949924

[69] Lévesque M, Potvin S, Marchand S, et al. Pain perception in schizophrenia: evidence of a specific pain response profile. *Pain Med*. 2012;13(12):1571–1579. doi:10.1111/j.1526-4637.2012.01505.x 23170852

[70] Urban-Kowalczyk M, Pigońska J, Śmigielski J. Pain perception in schizophrenia: influence of neuropeptides, cognitive disorders, and negative symptoms. *Neuropsychiatr Dis Treat*. 2015;11:2023–2031. doi:10.2147/NDT.S87666 PMC4532169 26273205

[71] Potvin S, Stip E, Tempier A, et al. Pain perception in schizophrenia: no changes in diffuse noxious inhibitory controls (DNIC) but a lack of pain sensitization. *J Psychiatr Res*. 2008;42(12):1010–1016. doi:10.1016/j.jpsychires.2007.11.001 18093615

[72] Russ MJ, Roth SD, Lerman A, et al. Pain perception in self-injurious patients with borderline personality disorder. *Biol Psychiatry*. 1992;32(6):501–511. doi:10.1016/0006-3223(92)90218-o 1445967

[73] Wongpakaran T, Wongpakaran N. Personality traits influencing somatization symptoms and social inhibition in the elderly. *Clin Interventions Aging*. 2014;9:157–164. doi:10.2147/CIA.S56246 PMC3894951 24477217

[74] Kanchanatawan B, Sirivichayakul S, Thika S, et al. Physio-somatic symptoms in schizophrenia: association with depression, anxiety, neurocognitive deficits and the tryptophan catabolite pathway. *Metab Brain Dis*. 2017;32(4):1003–1016. doi:10.1007/s11011-017-9982-7 28258445

[75] Aksu MH, Sahiner SY, Sahiner IV, et al. Relationship between somatization and psychiatric symptoms, especially anxiety, depression, alexithymia, and severity of addiction in male patients with alcohol and heroin addiction. *Dusunen Adam*. 2020;33(2):120–129. doi:10.14744/DAJPNS.2020.00071

[76] Drews A, Hazlett-Stevens H. Relationships between irritable bowel syndrome, generalized anxiety disorder, and worry-related constructs. *Int J Clin Health Psychol*. 2008;8(2):429–436.

[77] Hoehn-Saric R, McLeod DR, Zimmerli WD. Somatic manifestations in women with generalized anxiety disorder. Psychophysiological responses to psychological stress. *Arch Gen Psychiatry*. 1989;46(12):1113–1119. doi:10.1001/archpsyc.1989.01810120055009 2589925

[78] Joormann J, Stö Ber J. Somatic symptoms of generalized anxiety disorder from the DSM-IV: associations with pathological worry and depression symptoms in a nonclinical sample. *J Anxiety Disord*. 1999;13(5):491–503. 10.1016/s0887-6185(99)00017-1 10600050

[79] Farhoumandi N, Zarean M, Nasiri M, Jega M. Somatization mediates the structural relationship of alexithymia with anxiety and depression. *Iranian J Psych*. 2022;17(2):144–153. doi:10.18502/ijps.v17i2.8904 PMC9533350 36262762

[80] Pavony MT, Lenzenweger MF. Somatosensory processing and borderline personality disorder: pain perception and a signal detection analysis of proprioception and exteroceptive sensitivity. *Personality disord*. 2014;5(2):164–171. doi:10.1037/per0000017 23834516

[81] Gradinger T, Sack M, Cardinale V, et al. The glutamate to γ-aminobutyric acid ratio in the posterior insula is associated with pain perception in healthy women but not in women with borderline personality disorder. *Pain*. 2019;160(11):2487–2496. doi:10.1097/j.pain.0000000000001641 31339871

[82] Rosenbaum DL, White KS, Gervino EV. The impact of perceived stress and perceived control on anxiety and mood disorders in noncardiac chest pain. *J Health Psychol*. 2012;17(8):1183–1192. doi:10.1177/1359105311433906 22313669

[83] Berk E, Koçyiğit BF, Berk ŞŞ. The relationship between disease severity and body perception, mood disorder and quality of life in fibromyalgia syndrome. *Turkiye Klinikleri J Med Sci*. 2020;40(2):154–160. doi:10.5336/medsci.2019-72278

[84] Tull MT, Gratz KL, Salters K, Roemer L. The role of experiential avoidance in posttraumatic stress symptoms and symptoms of depression, anxiety, and somatization. *J Nerv Mental Dis*. 2004;192(11):754–761. doi:10.1097/01.nmd.0000144694.30121.89 15505519

[85] Masuda A, Tully EC. The role of mindfulness and psychological flexibility in somatization, depression, anxiety, and general psychological distress in a nonclinical college sample. *J Evidence-Based Complementary Alternative Med*. 2012;17(1):66–71. doi:10.1177/2156587211423400

[86] Masuda A, Mandavia A, Tully EC. The role of psychological inflexibility and mindfulness in somatization, depression, and anxiety among Asian Americans in the United States. *Asian Am J Psychol*. 2014;5(3):230–236. doi:10.1037/a0034437

[87] Jensen JC, Haahr JP, Frost P, Andersen JH. The significance of health anxiety and somatization in care-seeking for back and upper extremity pain. *Family Pract*. 2012;29(1):86–95. doi:10.1093/fampra/cmr046 21817088

[88] Granot M, Yovell Y, Somer E, et al. Trauma, attachment style, and somatization: a study of women with dyspareunia and women survivors of sexual abuse. *BMC Women’s Health*. 2018;18(1):29. doi:10.1186/s12905-018-0523-2 PMC5791220 29382322

[89] Boettger MK, Bär KJ. Perception for ischemic pain shows similarities in adjustment disorder and major depression. *Eur J Pain*. 2007;11(7):819–822. doi:10.1016/j.ejpain.2006.12.004 17239636

[90] Borlimi R, Riboli G, Nese M, Brighetti G, Vescovelli F. Altered pain perception, dietary restraint and drive for thinness in bulimia nervosa. *Psychiatry Res Commun*. 2023;3(2). doi:10.1016/j.psycom.2023.100113

[91] Brambila-Tapia AJLMLA, Macías Espinoza F, Reyes Domínguez YA, Ramírez García ML. Association of depression, anxiety. Stress and stress-coping strategies with somatization and number of diseases according to sex in the mexican general population. doi:10.3390/healthcare10061048

[92] Cruciani G, Zingaretti P, Lingiardi V, De Filippis S, Haggard P, Spitoni GF. The perception of pain, discriminative touch and affective touch in patients suffering from borderline personality disorder. *J Affective Disord*. 2023;341:185–193. doi:10.1016/j.jad.2023.08.126LK 37657618

[93] Faruqui Z, Meckley G, Jain A, Fluyau D. On the borderline of pleasure and pain: comorbid persistent genital arousal disorder and borderline personality disorder. *Prim Care Companion CNS Disord*. 2023;25(1). doi:10.4088/PCC.22cr03281LK 36638541

[94] Ghamari Kivi H, Jamshiddoust Miyanroudi F, Mousavi S, Ghavibazou E. Role of types of inner speech in the prediction of symptoms of anxiety, depression, somatization, and distress in the normal population. *J Pract Clin Psychol*. 2023;11(4):341–348. doi:10.32598/jpcp.11.4.807.2

[95] Kerber A, Gewehr E, Zimmermann J, et al. Adverse childhood experiences and personality functioning interact substantially in predicting depression, anxiety, and somatization. *Personal Ment Health*. 2023;17(3):246–258. doi:10.1002/pmh.1578 36740219

[96] Knopp M, Burghardt J, Oppenauer C, Meyer B, Moritz S, Sprung M. Affective and cognitive Theory of Mind in patients with alcohol use disorder: associations with symptoms of depression, anxiety, and somatization. *J Subst Use Abuse Treat*. 2024;157. doi:10.1016/j.josat.2023.209227 37992810

[97] Lee SJ, Lee M, Kim HB, Huh HJ. The relationship between interoceptive awareness, emotion regulation and clinical symptoms severity of depression, anxiety and somatization. *Psychiatry Invest*. 2024;21(3):255–264. doi:10.30773/pi.2023.0221 PMC10990629 38569583

[98] Li J, Li R, Li D, Zhang J, Luo X, Zhang Y. Serum BDNF levels and state anxiety are associated with somatic symptoms in patients with panic disorder. *Front Psychiatry*. 2023;14. doi:10.3389/fpsyt.2023.1168771 PMC10393281 37533888

[99] Lies J, Lau ST, Jones LE, Jensen MP, Tan G. Predictors and moderators of post-traumatic stress disorder: an investigation of anxiety sensitivity and resilience in individuals with chronic pain. *Ann Academy Med Singapore*. 2017;46(3):102–110. 28417134

[100] Meng J, Zhang T, Hao T, et al. Functional and structural abnormalities in the pain network of generalized anxiety disorder patients with pain symptoms. *Neuroscience*. 2024;543:28–36. doi:10.1016/j.neuroscience.2024.02.006 38382693

[101] Moran JK, Jesuthasan J, Schalinski I, et al. Traumatic life events and association with depression, anxiety, and somatization symptoms in female refugees. *JAMA Network Open*. 2023;6(7):E2324511. doi:10.1001/jamanetworkopen.2023.24511 PMC10359962 37471088

[102] Mor-Ben-Ishai S, Ben-Ezra M, Hamama-Raz Y, Leshem E, Levin Y. Subjective trauma perception as a distinctive predictor of somatization and disability during the 2022 Russian invasion of Ukraine. *J Psychiatr Res*. 2025;182:284–289. doi:10.1016/j.jpsychires.2025.01.027 39826379

[103] Nehme A, Moussa S, Fekih-Romdhane F, et al. Expressive suppression moderates the relationship between PTSD from COVID-19 and somatization and validation of the Arabic version of Patient Health Questionnaire-15 (PHQ-15). *PLoS One*. 2024;19(1). doi:10.1371/journal.pone.0293081 PMC10810523 38271356

[104] Toprak T, Ayribas B. A new perspective on the etiology of overactive bladder syndrome—could overactive bladder syndrome be associated with altered perception of somatic sensations as a result of obsessive-compulsive disorder? A case-control study. *Int Neurourology J*. 2025;29(1):48–55. doi:10.5213/inj.2448390.195 PMC12010897 40211838

[105] Trevisan DA, Mehling WE, McPartland JC. Adaptive and maladaptive bodily awareness: distinguishing interoceptive sensibility and interoceptive attention from anxiety-induced somatization in autism and alexithymia. *Autism Res*. 2021;14(2):240–247. doi:10.1002/aur.2458 33336935

[106] Verhaeghe P, Vanheule S, De Rick A. Actual neurosis as the underlying psychic structure of panic disorder, somatization, and somatoform disorder: an integration of Freudian and attachment perspectives. *Psychoanalytic Quarterly*. 2007;76(4):1317–1350. doi:10.1002/j.2167-4086.2007.tb00306.x 18085013

[107] Simon NM, Pollack MH, Tuby KS, Stern TA. Dizziness and panic disorder: a review of the association between vestibular dysfunction and anxiety. *Ann Clin Psych*. 1998;10(2):75–80. doi:10.1023/a:1026167000066 9669539

[108] Kumar V, Avasthi A, Grover S. Somatosensory amplification, health anxiety, and alexithymia in generalized anxiety disorder. *Indust psych j*. 2018;27(1):47–52. doi:10.4103/ipj.ipj_72_17 PMC6198604 30416291

[109] Terasawa Y, Oba K, Motomura Y, Katsunuma R, Murakami H, Moriguchi Y. Paradoxical somatic information processing for interoception and anxiety in alexithymia. *Eur J Neurosci*. 2021;54(11):8052–8068. doi:10.1111/EJN.15528 PMC9298728 34766398

[110] Nummenmaa L, Glerean E, Hari R, Hietanen JK. Bodily maps of emotions. *Proc Natl Acad Sci USA*. 2014;111(2):646–651. doi:10.1073/PNAS.1321664111/SUPPL_FILE/PNAS.201321664SI.PDF PMC3896150 24379370

[111] Folz J, Nikić K, Nikolić M, Janowicz V, Koelkebeck K, Kret ME. The role of the body in altered facial emotion perception in autism and social anxiety. *Behav Res Ther*. 2025;193:104819. doi:10.1016/j.brat.2025.104819 40712257

[112] Lyssenko L, Schmahl C, Bockhacker L, Vonderlin R, Bohus M, Kleindienst N. Dissociation in psychiatric disorders: a meta-analysis of studies using the dissociative experiences scale. *Ajp*. 2018;175(1):37–46. doi:10.1176/appi.ajp.2017.17010025 28946763

[113] Ducasse D, Courtet P, Olié E. Physical and social pains in borderline disorder and neuroanatomical correlates: a systematic review. *Curr Psychiatry Rep*. 2014;16(5):443. doi:10.1007/s11920-014-0443-2 24633938

[114] Krause-Utz A, Frost R, Chatzaki E, Winter D, Schmahl C, Elzinga BM. Dissociation in borderline personality disorder: recent experimental, neurobiological studies, and implications for future research and treatment. *Curr Psychiatry Rep*. 2021;23(6):37. doi:10.1007/s11920-021-01246-8 PMC8081699 33909198

[115] Zinchuk MS, Avedisova AS, Voinova NI, et al. Pain perception and nonsuicidal self-injurious behavior. *Z nevrol psikhiatr im SS Korsakova*. 2020;120(12):144. doi:10.17116/jnevro2020120121144 33459555

[116] Hanson MA, Barreiro PG, Crosetto P, Brockington D. The strain on scientific publishing. *Quantitative Sci Studies*. 2024;5(4):823–843. doi:10.1162/qss_a_00327

[117] Guo X, Lin F, Yang F, Chen J, Cai W, Zou T. Gut microbiome characteristics of comorbid generalized anxiety disorder and functional gastrointestinal disease: correlation with alexithymia and personality traits. *Front Psychiatry*. 2022;13. doi:10.3389/fpsyt.2022.946808 PMC9424665 36051551

[118] Engelen T, Solcà M, Tallon-Baudry C. Interoceptive rhythms in the brain. *Nat Neurosci*. 2023;26(10):1670–1684. doi:10.1038/s41593-023-01425-1 37697110

[119] Khalsa SS, Adolphs R, Cameron OG, et al. Interoception and mental health: a roadmap. *Biol Psych Cognitive Neurosci Neuroimag*. 2018;3(6):501–513. doi:10.1016/J.BPSC.2017.12.004 PMC6054486 29884281

[120] Schoeller F, Horowitz AH, Jain A, et al. Interoceptive technologies for psychiatric interventions: from diagnosis to clinical applications. *Neurosci Biobehav Rev*. 2024;156:105478. doi:10.1016/j.neubiorev.2023.105478 38007168

[121] Petzschner FH, Weber LAE, Gard T, Stephan KE. Computational psychosomatics and computational psychiatry: toward a joint framework for differential diagnosis. *Biol Psychiatry*. 2017;82(6):421–430. doi:10.1016/j.biopsych.2017.05.012 28619481

[122] Kouros I, Isaksson M, Ekselius L, Ramklint M. A cluster analysis of attachment styles in patients with borderline personality disorder, bipolar disorder and ADHD. *bord personal disord emot dysregul*. 2024;11(1):26. doi:10.1186/s40479-024-00271-2 PMC11523661 39472982

[123] Monsa R, Peer M, Arzy S. Self-reference, emotion inhibition and somatosensory disturbance: preliminary investigation of network perturbations in conversion disorder. *Eur J Neurol*. 2018;25(6):888–e62. doi:10.1111/ene.13613 29509290

[124] National Institute of Mental Health. Sensorimotor domain added to the RDoC framework. 2019. Available form: https://www.nimh.nih.gov/news/science-updates/2019/sensorimotor-domain-added-to-the-rdoc-framework. Accessed September 25, 2026.

[125] Lobo-Escolar A, Saz P, Marcos G, Quintanilla MÁ, Campayo A, Lobo A. Somatic and psychiatric comorbidity in the general elderly population: results from the ZARADEMP project. *J Psychosomatic Res*. 2008;65(4):347–355. doi:10.1016/j.jpsychores.2008.03.002 18805244

[126] Kaplan CM, Kelleher E, Irani A, Schrepf A, Clauw DJ, Harte SE. Deciphering nociplastic pain: clinical features, risk factors and potential mechanisms. *Nat Rev Neurol*. 2024;20(6):347–363. doi:10.1038/s41582-024-00966-8 38755449

[127] Nagy C, Jones P, Massett HA, Kelley AS. National Institute on Aging efforts toward ensuring representation of diverse older adults in clinical studies. *J Am Geriatrics Soc*. 2024;72(9):2907–2911. doi:10.1111/jgs.18831 PMC11445706 38445863

[128] Molinard-Chenu A, Chytas V. Bodily symptoms in psychiatry: a mechanistic scoping review (Psycinfo). 2025. doi:10.1079/searchRxiv.2025.01192 PMC13640999 42841023

[129] Molinard-Chenu A, Chytas V. Bodily symptoms in psychiatry: a mechanistic scoping review (Pubmed); 2025. doi:10.1079/searchRxiv.2025.01190 PMC13640999 42841023

[130] Molinard-Chenu A, Chytas V. Bodily symptoms in psychiatry: a mechanistic scoping review (Embase); 2025. doi:10.1079/searchRxiv.2025.01191 PMC13640999 42841023

